# Interobserver variability and accuracy of p16/Ki-67 dual immunocytochemical staining on conventional cervical smears

**DOI:** 10.1186/s13000-019-0821-5

**Published:** 2019-05-24

**Authors:** Veronika Kloboves Prevodnik, Tine Jerman, Nataša Nolde, Alenka Repše Fokter, Sandra Jezeršek, Živa Pohar Marinšek, Ulrika Klopčič, Simona Hutter Čelik, Kristina Gornik Kramberger, Maja Primic Žakelj, Urška Ivanuš

**Affiliations:** 1 Department of Cytopathology, Institute of Oncology, Zaloška 2, SI-1000 Ljubljana, Slovenia; 2 ZORA National Cervical Cancer Screening Programme and Registry, Epidemiology and Cancer Registry, Institute of Oncology, Ljubljana, Slovenia; 3 Department of Pathology and Cytology, Celje General Hospital, Celje, Slovenia; 4 Department of Pathology and Cytology, General Hospital Izola, Izola, Slovenia; 5 Department of Pathology, University Medical Centre, Maribor, Slovenia

**Keywords:** Cervical cancer screening, p16/Ki-67, Immunocytochemical staining, Agreement, Diagnostic accuracy

## Abstract

**Background:**

p16/Ki-67 dual immunocytochemical staining (DS) has been proven as a sensitive and specific test for triage of HPV positive women with good reproducibility and accuracy. However, implementation of the test into an organized screening program (OSP) is not easy. The aims of this study were to compare the performance and agreement of DS results among three Slovenian cytopathological laboratories involved in the national OSP, and to define cases where staining results can be difficult to interpret.

**Methods:**

Cervical smears were obtained for DS from 129 women referred to colposcopy. Smears were evaluated blindly in three laboratories by a cytotechnologist and a cytopathologist after initial training. Results were positive, suspicious, negative or inadequate. Five characteristics of DS staining were recorded. After primary evaluation, an extensive expert-led additional training was undertaken, including a discussion of difficult cases and a practical exam. Smears were re-evaluated and results compared to primary evaluation.

**Results:**

After the additional training, the overall percentage of agreement among the three laboratories increased from 77.5 to 89.9% and kappa increased from 0.70 to 0.86. Sensitivity for CIN2+ increased in two laboratories, to 90.5 and 85.7%, without the loss of specificity (75.8%). In one laboratory, the sensitivity slightly decreased from 90.5 to 88.9%, but the specificity increased from 63.6 to 68.2%. Difficult cases had significantly less DS cells, weak intensity of p16 staining, suboptimal cell morphology and background staining compared to positive cases.

**Conclusion:**

Additional expert-led training and discussion of difficult cases are necessary for accurate interpretation of DS in laboratories involved in OSP. The most difficult cases were those with single stained cells and weak p16 staining. Training protocol for safe implementation of p16/Ki-67 DS in OSP is proposed.

**Electronic supplementary material:**

The online version of this article (10.1186/s13000-019-0821-5) contains supplementary material, which is available to authorized users.

## Introduction

p16/Ki-67 dual immunocytochemical staining (DS) has been confirmed as a sensitive and specific triage test for HPV positive women [1] as well as for women with low-grade cytology [2], as a potential screening test for cervical cancer [3] and as a test for surveillance after treatment of high-grade squamous intraepithelial lesions (cervical intraepithelial neoplasia grade 2 or worse (CIN2+)) [4]. Literature describes this test as easy to interpret compared to the p16^INK4a^ test because it avoids the need for morphological evaluation. The threshold for a positive test is a single dual-stained p16/Ki-67 cell on the slide [5, 6]. Recently, five studies have confirmed good reproducibility of p16/Ki-67 DS [6–10]. Their findings support the implementation of DS into organized, HPV-based cervical cancer screening programs as a triage test, even in settings where expert staff is not available.

The drawback of HPV based screening is its lower specificity for CIN2+ compared to cytology, due to a higher prevalence of HPV infection compared to the prevalence of cervical precancerous lesions and cancer. To overcome this, additional triage tests were designed to identify women who should be referred to colposcopy [11]. Wright et al. have recently presented the results of a sub-study nested into the Athena trial. The authors demonstrated that DS triage had higher sensitivity and similar specificity compared to cytology, in a group of 3467 high-risk HPV-positive women. The specificity was similar for DS and cytology triage [12]. On the other hand, Ebisch et al. [13], in the subset of the PROHTECH 3B study, showed that DS triage for high-risk HPV positive women had a higher specificity and similar sensitivity compared to cytology.

In Slovenia, an organized, conventional cytology-based national wide cervical cancer screening program, ZORA, was implemented in 2003, with 3-year screening intervals in the 20–64 age group. Since the implementation of the organized screening program (OSP), the age standardized incidence rate (world standard) decreased from 15.3/100,000 in year 2003 to 7.8/100,000 in year 2016 [14]. Since Slovenia is considering a change in national screening policy in the future, we have conducted a study to become familiar with DS technology, and to test the feasibility and performance of this test for the first time in the Slovenian setting.

The aims of this study were (1) to compare the performance of DS and agreement of the results obtained in DS interpretation among three Slovenian cytopathological laboratories involved in the national OSP ZORA and (2) to define characteristics of DS which can make the test difficult to interpret.

## Material and methods

### Study design

The study population included 129 women who underwent colposcopy at Celje General Hospital or at University Medical Centre Maribor between April and August 2014. According to the Slovenian national guidelines they were invited to colposcopy either due to high-grade (HG) cytology, an HPV-positive triage test after low-grade pathological changes or due to a positive HPV test during follow-up after treatment of high-grade CIN [15]. Pregnant women and women with acute vaginitis or cervicitis were not included in the study. Gynecologists collected one smear for cytology and DS (spilt sample technique). Women with abnormal colposcopy underwent colposcopy guided biopsy, followed by histological evaluation, including p16 immunohistochemical staining test, according to WHO recommendations [16, 17].

Three Slovenian laboratories participated in the study: Institute of Oncology (LAB1), Celje General Hospital (LAB2), and University Medical Centre Maribor (LAB3). All cytotechnologists (thirteen) and cytopathologists (seven) employed in these laboratories were participating.

The reading of DS slides was divided into primary reading of all slides after initial training and the secondary reading of slides with discrepant results after additional training. In each laboratory slides were distributed among cytotechnologists who examined them first, spotted the DS cells and passed them on to a cytopathologist. Both results were recorded and therefore, each case had six readings. Difficult cases with discrepant results between cytotechnologist and cytopathologist were discussed at a multi-head microscope in each laboratory and a consensus result was reached. Results were evaluated after the primary reading. They were considered consistent when all six readings were the same. When at least one reading differed from the others, or if the number of positive cells differed among the six readings, the result was inconsistent. The slides with inconsistent results were first re-evaluated in a consensus meeting by the five out of seven cytopathologists participating in the study (reference1 results). After that, a troubleshooting slide review was organized with the manufacturer. All participants of the study were present, the multi-head microscope with video projection was used, and a new consensus result was reached (reference2 result). Four months after primary evaluation, DS slides with inconsistent results were evaluated for a second time in the same way as in the primary evaluation. We assumed that re-examination of consistent DS results would not be beneficial due to the high probability of the same results.

### Immunocytochemical p16/Ki-67 dual staining and interpretation

DS was performed on conventional smears fixed with Merckofix spray (MERCK) and transported to the LAB1, where automated CINtec PLUS cytology test (Roche) was performed in Benchmark (Ventana Roche) immunostainer. DS slides were interpreted blindly with respect to cytology, histology and clinical data in all three laboratories.

DS was interpreted as positive or negative according to the recommendation of the manufacturer. The test was positive when both p16 and Ki-67 staining were observed in at least a single cell. Negative cases without positive internal control for p16/Ki-67 staining were considered inadequate. For the purpose of the study we have introduced a suspicious category to identify cases which were difficult to interpret. All participants recorded the number of positive or suspicious cells (one to five), p16 and Ki-67 staining intensity (appropriate or weak), cell morphology (preserved or less preserved), counter stain (appropriate or weak) and background staining (not present/weak or strong).

### Training design

Cytotechnologists and cytopathologists had no previous experience with DS interpretation. The initial training program for DS interpretation included lectures and discussion of 40 teaching slides with the manufacturer in the form of tele-cytology. Training was completed in 4 h. After primary evaluation in all three laboratories, additional training took place, also provided by the manufacturer. This time training lasted two half days. It included a troubleshooting slide review of discordant cases, lectures and a slide seminar in which participants examined slides under the microscope. Training was concluded with an exam which all the participants passed.

### Study outcomes and statistical analysis

*The primary outcomes* were: (1) agreement in DS interpretation between the three laboratories, as well as between cytotechnologists, cytopathologists and both references; (2) accuracy prior to and after the additional training. Agreement was evaluated with overall percentage agreement (OPA), Cohen’s kappa and McNemar’s test *p*-value (for laboratory pairs and laboratory-reference pairs) and Light’s (summary) kappa and Cochran’s Q test *p*-value (for all three laboratories). For interpretation of kappa values we used the following scale: below 0.20 (poor), 0.21–0.40 (fair), 0.41–0.60 (moderate), 0.61–0.80 (good) and > 0.81 (very good) [18]. Accuracy of DS results was evaluated with sensitivity, specificity, positive (PPV) and negative predictive value (NPV) of DS for CIN2+ diagnosed within 1 year after enrolment. If a woman had a negative colposcopy and no histology result in a 1-year follow-up, she was considered as negative for disease. For the purpose of these analyses, the suspicious DS results were considered positive, and inadequate as negative. In analyses that represent laboratory results, consensus diagnosis between cytopathologist and cytotechnologist was included in the calculations.

*The secondary outcomes* were related to resolving difficult cases. We wanted to discern how staining characteristics contributed to difficulty in DS interpretation. In these analyses, each positive and suspicious DS interpretation result for various staining characteristics was interpreted as an independent result, and was included in the calculation. For the assessment of whether the difference between positive and suspicious interpretation results was significant, the Mann-Whitney test was used for ordinal (cells number), while chi-square (p16 staining intensity and background staining) and Fisher’s exact test (for expected cell counts < 5; Ki-67 staining intensity, cell morphology and counter stain) were used for nominal dependent variables.

All analyses were conducted with SPSS v22.0 [19] and R v3.5.1 [20], using 2-tailed tests and the significance level α = 0.050.

## Results

### Patients’ characteristics

The average age of the 129 women in the study was 36.8 years (standard deviation: 11.1, range: 20–62). Fifty percent of the women were older than 35 years. Forty-seven percent (60/129) of the women had cytological diagnosis of high-grade squamous intraepithelial lesion or worst (HSIL+) in the last year prior to inclusion in the study. Tissue for histologic evaluation was obtained in 77% (99/129) of women. Forty-nine percent (63/129) of women had histologically proven CIN2+, and 26% (33/129) had low-grade squamous intraepithelial lesions (CIN1) within 1 year after inclusion in the study.

### Results of p16/Ki-67 dual staining

Thirty-nine percent (50/129) of DS results were consistent and 61% (79/129) were inconsistent after initial training. Sixty differed in test result and 39 in the number of positive cells. After additional training, 37% (49/129) of results remained inconsistent, 24 differed in test result, and 25 in number of positive cells. When the number of positive cells was excluded from the evaluation, the percentage of consistent DS results increased from 53 to 81% after additional training.

After additional training, the number of suspicious cases decreased by 7.0–11.6 percentage points in the three laboratories, while the number of positive cases increased by 5.5–14.7 percentage points (Table 1). The number of negative cases increased only in LAB2. No CIN2+ was present among the suspicious cases from two laboratories, however, one CIN2+ was present among the suspicious cases from one laboratory. Compared to results after initial training, less CIN2+ were missed within negative cases in two laboratories after additional training, while one additional CIN2+ was missed in one laboratory. No CIN2+ was found within the inadequate DS result category.

**Table 1:** p16/Ki-67 study results and CIN2+ outcome for three cytopathology laboratories and references

Reviewer	Categories of p16/Ki-67 dual staining result	Initial training		Additional training	
		p16/Ki-67 dual staining result N (%)^a^	CIN2+ outcome N (PV, %)^b^	p16/Ki-67 dual staining result N (%)^a^	CIN2+ outcome N (PV, %)^b^
LAB1	positive	60 (46.5)	46 (76.7)	72 (55.8)	57 (79.2)
	suspicious	10 (7.8)	6 (60.0)	1 (0.8)	0 (0.0)
	negative	58 (45.0)	11 (19.0)	56 (43.4)	6 (10.7)
	unsatisfactory	1 (0.8)	0 (0.0)	0 (0.0)	0 (0.0)
LAB2	positive	67 (51.9)	51 (76.1)	74 (57.4)	56 (75.7)
	suspicious	14 (10.9)	6 (42.9)	3 (2.3)	0 (0.0)
	negative	40 (31.0)	6 (15.0)	52 (40.3)	7 (13.5)
	unsatisfactory	8 (6.2)	0 (0.0)	0 (0.0)	0 (0.0)
LAB3	positive	49 (38.0)	38 (77.6)	68 (52.7)	53 (77.9)
	suspicious	17 (13.2)	12 (70.6)	2 (1.6)	1 (50.0)
	negative	63 (48.8)	13 (20.6)	58 (45.0)	9 (15.5)
	unsatisfactory	0 (0.0)	0 (0.0)	1 (0.8)	0 (0.0)
Reference1	positive	60 (46.5)	49 (81.7)		
	suspicious	12 (9.3)	5 (41.7)		
	negative	57 (44.2)	9 (15.8)		
	unsatisfactory	0 (0.0)	0 (0.0)		
Reference2	positive			74 (57.4)	56 (75.7)
	suspicious			1 (0.8)	0 (0.0)
	negative			54 (41.9)	7 (13.0)
	unsatisfactory			0 (0.0)	0 (0.0)

LAB1 … Laboratory1, LAB2 … Laboratory2, LAB3 … Laboratory3, Reference1 … consensus of 5 cytopathologists, Reference2 … consensus obtained during discussion between participants and expert

^a^Consensus results within each laboratory/reference (*n* = 129)

^b^PV...predictive value (number of CIN2 + detected within specific category of p16/Ki-67 dual staining result divided by the number of test results in specific category)

### Agreement between the laboratories and reviewers

Agreement of DS results between the laboratories improved after the additional training (Table 2). Agreement between all three laboratory pairs was good after initial training and very good after additional training as evidenced by higher OPA and kappa values. Similar improvement was observed when the DS results were compared between all three laboratories (Table 2).

**Table 2:** Agreement of p16/Ki-67 results

	Initial training			Additional training		
	OPA	*p* value	Kappa (95% CI)	OPA	p value	Kappa (95% CI)
LAB1-LAB2	86.8	0.015	0.73 (0.61–0.85)	93.8	0.289	0.87 (0.79–0.96)
LAB1-LAB3	86.0	0.480	0.72 (0.60–0.84)	93.0	0.505	0.86 (0.77–0.95)
LAB2-LAB3	82.2	0.004	0.64 (0.51–0.77)	93.0	0.046	0.86 (0.77–0.95)
LAB1-LAB2-LAB3	77.5	0.002	0.70 (0.59–0.78)	89.9	0.058	0.86 (0.78–0.92)

OPA … Overall percent agreement, LAB1 … Laboratory1, LAB2 … Laboratory2, LAB3 … Laboratory3

Furthermore, agreement of DS results between each of the six groups of evaluators (cytotechnologists and cytopathologists from three laboratories) and reference2 also improved after the additional training (Additional file 1: Table S1). OPA and kappa values were higher after additional training for all reviewer groups (OPA range: 90.7%–96.9% compared to 86.0%–89.9%; kappa range: 0.81–0.94 compared to 0.70–0.80). Agreement between reference1 and reference2 was very good (OPA was 96.1% and kappa value 0.92). Summary kappa value before the additional training was 0.68 (95% CI: 0.57–0.77) for cytotechnologists and 0.70 (95% CI: 0.59–0.78) for cytopathologists. After the additional training, summary kappa value increased to 0.78 (95% CI: 0.68–0.85) for cytotechnologists and 0.86 (95% CI: 0.78–0.92), for cytopathologists.

### Accuracy of p16/Ki-67 results

**Fig. 1:**
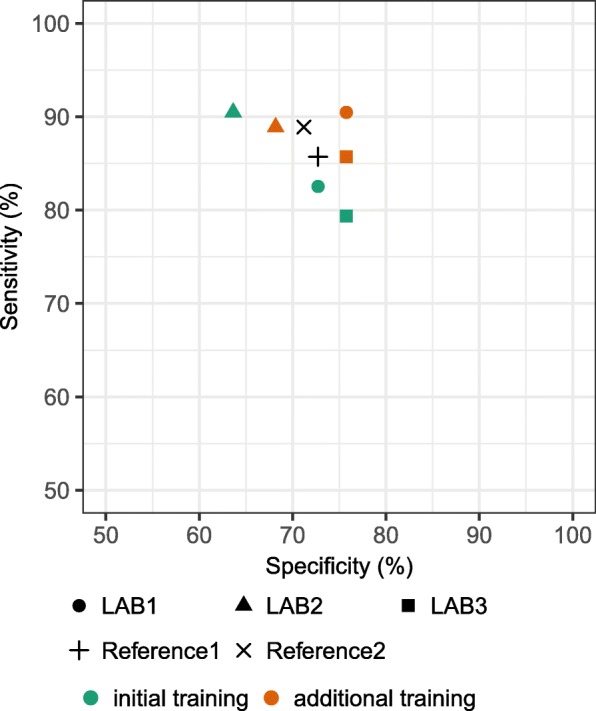
Sensitivity and specificity of p16/Ki-67 DS for detecting CIN2+ LAB1 … laboratory1, LAB2 … laboratory2, LAB3 … laboratory3, Reference1 … consensus of 5 cytopathologists, Reference2 … consensus obtained during discussion between participants in the study and expert

CIN2+ sensitivity was higher after the additional training in two out of three laboratories, without decrease in specificity. In the laboratory which had the highest sensitivity and the lowest specificity after the initial training (LAB2), the sensitivity slightly decreased after the additional training on behalf of a large increase in specificity (Additional file 1: Table S2). After the additional training, the highest sensitivity and specificity were obtained in LAB1. These results were even better than the results of reference2 (Fig. 1).

The differences in sensitivity and specificity between cytotechnologists/cytopathologists and reference2 after the additional training were mostly smaller than after initial training. After additional training, accuracy was mostly better than reference1 evaluation, and similar to the reference2 evaluations (Additional file 1: Table S1).

### Staining characteristics of difficult cases

**Fig. 2:**
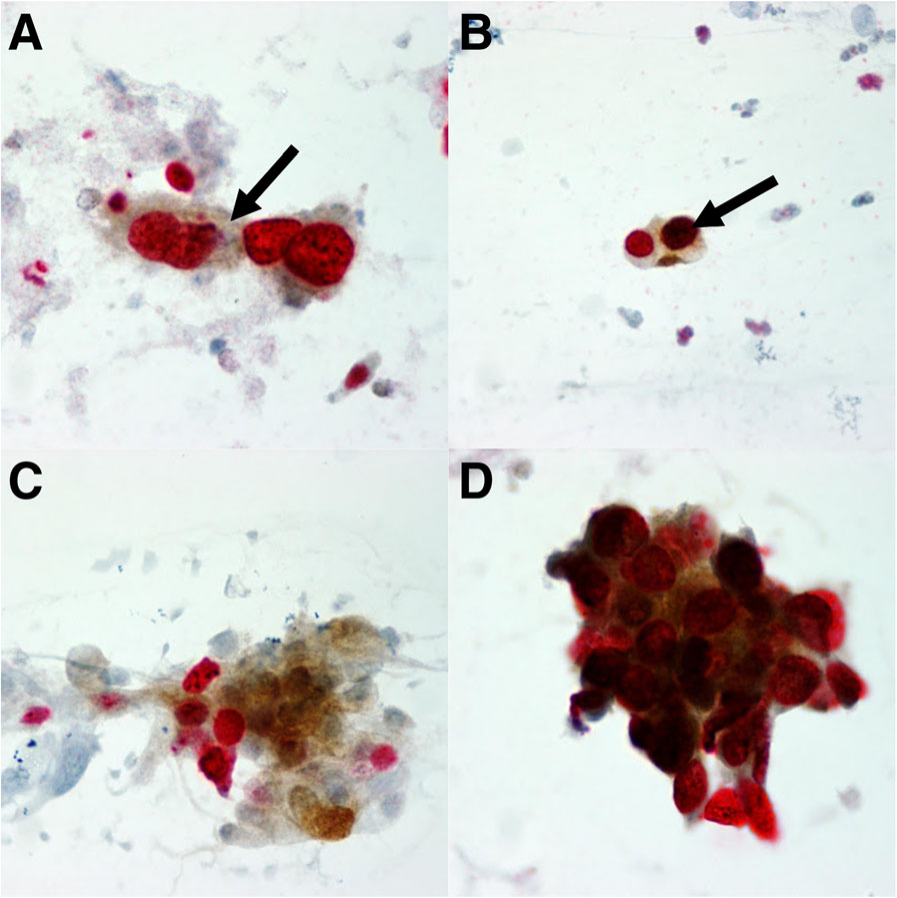
A few cases where p16/Ki-67 DS was difficult to interpret. **a** Weak p16 staining of the cytoplasm in a case where even the expert was not sure whether the test could be called positive. **b** Arrow points to a nucleus where it is difficult to differentiate intense p16 from Ki-67 staining. **c** A group of cells designated as suspicious for p16/Ki-67 DS by the cytotechnologists/cytopathologists and by the expert (reference2). **d** A group of cells where it is difficult to decide whether the p16/Ki-67 DS can be called positive

We found a statistically significant difference in the number of p16/Ki-67 DS cells, p16 staining intensity, cell morphology and background staining between positive and suspicious DS interpretation results (Table 3, Fig. 2). The number of stained cells was significantly higher in positive DS interpretation results compared to the suspicious ones. Over 50% of positive results had five or more positive cells, contrary to suspicious ones where 45% had only one suspicious cell. Weak p16 staining was significantly more common in suspicious results compared to the positive ones. Less preserved cell morphology and stronger background staining were more commonly observed in suspicious compared to positive DS interpretation results. Weaker counter staining was also more commonly observed in suspicious compared to positive DS interpretation results, but the difference was not statistically significant.

**Table 3:** Characteristics of p16/Ki-67 dual staining in suspicious and positive recordings

p16/Ki-67 staining characteristics		p16/Ki-67 result				*p*-value
		positive		suspicious		
		N	%	N	%	
Number of stained cells	1	57	16.1	36	45.0	0.000
	2	31	8.8	18	22.5	
	3	37	10.5	13	16.3	
	4	17	4.8	5	6.3	
	5	211	59.8	8	10.0	
p16 staining intensity	appropriate	238	82.6	35	63.6	0.001
	weak	50	17.4	20	36.4	
Ki-67 staining intensity	appropriate	273	94.8	53	96.4	1.000
	weak	15	5.2	2	3.6	
Cell morphology	preserved	285	99.3	51	92.7	0.007
	less preserved	2	0.7	4	7.3	
Counter stain	appropriate	284	99.0	52	94.5	0.055
	weak	3	1.0	3	5.5	
Background staining	not present/ weak	252	88.1	43	78.2	0.048
	strong	34	11.9	12	21.8	

Table includes staining characteristics from positive and suspicious readings recorded by all participants before additional training. Some results had missing data on staining characteristics, therefore sums for individual characteristics differ

## Discussion

The results of our study demonstrated that interpretation of p16/Ki-67 DS is not a very simple task since there were 61% discordant results between three laboratories after initial training. After additional training, the number of discordant results decreased and agreement in DS interpretation between reviewers increased from good to very good. Clinical accuracy in detecting CIN2+ increased in three laboratories mostly due to an increase in sensitivity. Furthermore, we demonstrated that characteristics of DS strongly influence result interpretation.

We are aware of four published papers and one conference presentation reporting on studies similar to ours [6–10]. All five studies were investigating agreement between DS reviewers and three also reported on clinical accuracy. Two reports mentioned reasons for difficulties in interpretation of certain cases [8, 9]. However, there are many differences in methodology among these reports as well as in regard to our study and comparison is possible to a limited extent. When we compare kappa values across different studies and settings, as a measure of agreement, one should be careful, since these values are dependent on the prevalence of the disease [21], and can also be biased due to a difference in study design or data preparation. However, the consistency of results and patterns across different studies may imply that these results are relevant and causative [22].

In McMenamin’s study the kappa values for agreement between p16/Ki-67 DS reviewer pairs were within the category of “very good” [9]. The summary kappa values reported in Wentzensen’s [6], Allia’s [7] and Benevolo’s [8] studies all correspond to the category of good agreement between DS readers since they range from 0.61 to 0.71. These figures are similar to the summary kappa values of 0.68 for cytotechnologists and 0.70 for cytopathologists obtained in our study before additional training. It is interesting that summary kappa results are so much alike despite differences in methodology. We performed the test on conventional smears while in the other four studies DS test was performed on liquid-based preparations; in McMenamin’s [9], Wentzensen’s [6], Allia’s [7] study only two result categories were used while in the Benevolo’s [8] and in our study, an inconclusive/suspicious and an inadequate category were introduced in addition to the positive and negative ones. However, in our study four results categories were used only in recording DS results but not in agreement calculations. Other differences were the number of specimens (42, 129, 480, 500, 972), the number of reviewers (3, 7, 10–11, 17–22, 20); the number of laboratories (1–9), the number of reports per slide (2–7), and the level of training in cytology as well as in p16/Ki-67 DS interpretation. Since all initial training was provided by the same manufacturer we can assume that it was more or less the same. However, in Benevolo’s [8] study some of the evaluators were experienced in DS evaluation, while in Allia’s [7] study a multi-head microscope session of discrepant cases was organised after evaluators reviewed 150 slides. During our study, difficult cases were also discussed within each laboratory. Since agreement results in the four studies were so similar despite methodological differences we agree with the conclusion of Benevolo et al. [8] that p16/Ki-67 DS is a robust test. As such it can be considered for the use in cervical cancer screening programs.

Even though our results on agreement between laboratories after initial training were comparable to those reported in the literature we were not satisfied to conclude that initial training was sufficient for safe implementation of DS test into the national cancer OSP. After the additional training, the summary kappa value for the three laboratories participating in our study improved, although the difference was not statistically significant. Improvement was observed in the subgroups of cytotechnologists as well as in the subgroups of cytopathologists. An improvement of kappa values after training was also observed in Allia’s study, where non-experts’ kappa values increased from 0.56 (95% CI = 0.46–0.67) to 0.75 (95% CI = 0.68–0.80), and experts’ kappa values increased from 0.63 (95% CI = 0.55–0.72) to 0.73 (95% CI = 0.67–0.78) [7]. Furthermore, in Benevolo’s study, lower kappa values were observed with inexperienced readers compared to experienced ones; the corresponding summary kappa values were 0.50 (95% CI = 0.39–0.62) and 0.75 (95% CI = 0.64–0.84) respectively [8]. In view of the above mentioned results we believe that proper training and experience are important determinates of agreement in DS reading.

Wentzensen et al. concluded that: “Implementation of p16/Ki-67 cytology evaluation is feasible in routine cytology laboratories with limited training” [6]. The conclusion was based on the comparison of their accuracy results of the DS test between cytotechnologists and the reference evaluations. The sensitivity and specificity estimates of DS test for detection of CIN2+ among their study population of HPV positive women were similar between the two evaluations (sensitivity: 82% vs 84%; specificity: 63.9% vs 62.5%). Similar accuracy results were also obtained by Allia et al., where results of non-expert readers differed by only a few percentage points from the results of expert readers [7]. Therefore, Allia et al. also concluded that: “After a short training phase, the interpretation of dual staining could be performed even by staff not trained in the morphological interpretation of cytology” [7]. The accuracy outcome in our study was very similar to the above mentioned results. However, despite the additional training, there were still differences in the performance of the DS evaluators. Some suspicious cases still remained after additional training and 37% of DS results remained inconsistent. Such an outcome points to the fact that evaluators had many difficulties in reading certain slides.

We have identified two main characteristics of difficult p16/Ki-67 DS smears that might lead to lower agreement and accuracy of the test: (1) low number of dual stained cells; and (2) weak p16 staining. (Fig. 2a) It has already been recognized that low number of dual stained cells could interfere with the accuracy of the results [6, 8, 9]. In the study of Wentzensen et al. 48% of cytotechnologists called cases negative when just one dual stain-positive cell was present whereas “9% called a case positive when the reference evaluation did not detect any dual stain-positive cells” [6]. In the study of Benevolo et al. there were 26% of slides with low level of agreement and these mostly had less than five double stained cells [8]. Weak p16 staining may be more problematic in staff familiar with immunocytochemical reading of fine needle biopsies, because there, weak staining is usually considered unspecific. Poorly preserved cell morphology and strong background staining also contributed to difficult interpretation in our study since they were significantly more common in suspicious compared to positive readings. Weak p16 staining and poorly defined cell borders in cell clusters were also observed by Benevolo et al. [8] as well as by McMenamin et al. [9]. The cytotechnologists in McMenamin’s study also reported that interpretation of cell groups was sometimes challenging and that there was “occasional difficulty in differentiating intense p16 nuclear staining from Ki-67 staining” [9]. The interpreters of DS in our study also observed the above mentioned difficulties, however, they were not systematically recorded (Fig. 2b-d).

One of the important strengths of our study was that all women underwent colposcopy and biopsy followed by histological examination in case of an abnormal colposcopy result. Therefore, we were able to monitor the learning progress with two sets of indicators: agreement and accuracy. Agreement indicators alone are not sufficient for monitoring, since the accuracy of the test could be low despite excellent agreement. Another advantage was the setting of the study within the OSP. Firstly, three out of nine Slovenian laboratories became proficient at DS reading and laboratory expertise was built. Secondly, slides with discordant results were discussed on a multi-head microscope within each laboratory. With this we simulated a regular screening setting, and possibly improved accuracy measures. Slide discussion was probably also the reason that agreement for all three laboratories before additional training was higher in our study compared to results in some published reports. Third, the participation of three laboratories with a different number of employed cytopathologists and different experience with immunostaining (other than DS) gave us the opportunity to observe differences in their results that could be associated with these determinants.

The limitation of our study was that we could not assess the intra-laboratory agreement between cytotechnologists and cytopathologists, since slides were discussed internally. However, the design of the study allowed us to compare the results between categories of evaluators (cytotechnologists and cytopathologists) among three laboratories. Evaluators could see the same slide more than once, which could bias the results. However, since there were more evaluators in each laboratory, not all slides were seen by each evaluator. Moreover, we overcame this limitation with the 4-month long time gap between the first and second readings of the slides. Only one cytopathologist saw all slides twice. Despite that, that laboratory had the lowest specificity and medium sensitivity after additional training. This indicates that seeing the same slide more than once did not produce a relevant bias.

## Conclusion

In countries without expertise in DS reading, the implementation of the test may be difficult despite the extensive experience with the cytology screening program, such as in Slovenia. Our results suggest that training based on lectures and examination of teaching slides is not sufficient for the safe implementation of DS in an OSP. Additional expert-led training and discussion of difficult cases are necessary for accurate interpretation.

Based on the experience from our study, we propose a training program where lectures and examination of teaching slides are followed by the individual, blinded evaluation of at least 100 DS stained slides from a high-risk population. An experienced senior cytotechnologist should discuss the slides with discordant results with the student at the multi-head microscope. For difficult cases, consultations with an expert should be available. After the training is completed, evaluation should be carried out to compare the student’s results (agreement as well as accuracy) to reference results (which should be available for the set of learning slides). At the end of the training, a student must pass a practical exam, on a set of slides that consist of difficult as well as easy cases. During the training, the suspicious category of results should be available to the student, and all suspicious cases should be resolved by personal discussion between the student and the expert. Such a training program is also suitable for laboratories with staff inexperienced in DS reading.

## Additional file

Additional file 1: **Table S1.** Individual comparison of p16/Ki-67 agreement and performance between cytoscreeners, cytopathologists and reference2, and between reference1 and reference2, before and after the additional training. **Table S2.** Sensitivity, specificity, negative and positive predictive value of p16/Ki-67 DS results for detecting CIN2+ in three cytopathology laboratories, for references1 and reference2 before and after the additional training. (DOCX 19 kb)

## Abbreviations

CIN2 +: Cervical intraepithelial neoplasia grade 2 or worse
DS: Dual immunocytochemical staining
HG: High-grade
NPV: Negative predictive value
OPA: Overall percentage agreement
OSP: Organized screening program
PPV: Positive predictive value
