# Supplementary material for: Interobserver variability and accuracy of p16/Ki-67 dual immunocytochemical staining on conventional cervical smears
Source: Diagn Pathol. 2019 May 24;14:48. doi: 10.1186/s13000-019-0821-5 (PMC6533697; doi:10.1186/s13000-019-0821-5)
Supplement: Supplementary file 1 — Table S1. Individual comparison of p16/Ki-67 agreement and performance between cytoscreeners, cytopathologists and reference2, and between reference1 and reference2, before and after the additional training. Table S2. Sensitivity, specificity, negative and positive predictive value of p16/Ki-67 DS results for detecting CIN2+ in three cytopathology laboratories, for references1 and reference2 before and after the additional training. (DOCX 19 kb) [file 13000_2019_821_MOESM1_ESM.docx]

***Table S1.*** Individual comparison of p16/Ki-67 agreement and performance between cytoscreeners, cytopathologists and reference2, and between reference1 and reference2, before and after the additional training.

| **Training** | **Reviewer** |  | **OPA** | **p value** | **Kappa (95% CI)** | **Difference in sensitivity*** | **Difference in specificity*** |
| --- | --- | --- | --- | --- | --- | --- | --- |
| Initial | Cytotechnologists1 |  | 89.1 | 0.061 | 0.78 (0.67-0.89) | 7.9 | -4.5 |
|  | Cytotechnologists2 |  | 86.0 | 0.010 | 0.70 (0.58-0.83) | -1.6 | 16.7 |
|  | Cytotechnologists3 |  | 86.8 | 0.146 | 0.73 (0.62-0.85) | 7.9 | -3.0 |
|  | Cytopathologists1 |  | 89.9 | 0.267 | 0.80 (0.69-0.90) | 6.3 | -1.5 |
|  | Cytopathologists2 |  | 89.1 | 0.181 | 0.77 (0.66-0.89) | -1.6 | 7.6 |
|  | Cytopathologists3 |  | 86.8 | 0.052 | 0.74 (0.62-0.85) | 9.5 | -4.5 |
| Additional | Cytotechnologists1 |  | 91.5 | 1.000 | 0.83 (0.73-0.92) | 0.0 | -1.5 |
|  | Cytotechnologists2 |  | 95.3 | 0.683 | 0.90 (0.83-0.98) | 1.6 | 4.5 |
|  | Cytotechnologists3 |  | 90.7 | 0.773 | 0.81 (0.71-0.91) | 0.0 | -3.0 |
|  | Cytopathologists1 |  | 95.3 | 0.683 | 0.90 (0.83-0.98) | -1.6 | -4.5 |
|  | Cytopathologists2 |  | 96.9 | 0.617 | 0.94 (0.87-1.00) | 0.0 | 3.0 |
|  | Cytopathologists3 |  | 93.0 | 0.182 | 0.86 (0.77-0.95) | 3.2 | -4.5 |
| Reference | Reference1 |  | 96.1 | 0.371 | 0.92 (0.85-0.99) | 3.2 | -1.5 |
|  | **Reference2** |  | **100.0** |  | **1.00** | **0.0** | **0.0** |

*OPA…overall percent agreement, Cytotechnologists1…cytotechnologists from LAB1, Cytotechnologists2…cytotechnologists from LAB2, Cytotechnologists3…cytotechnologists from LAB3, Cytopathologists1…cytopathologist from LAB1, Cytopathologists2…cytopathologists from LAB2, Cytopathologists3…cytopathologists from LAB3, Reference1…consensus of 5 cytopathologists, Reference2…consensus obtained during discussion between participants of the study and expert.*

*** Difference in CIN2+ sensitivity and specificity were calculated as an estimation for reference2 minus individual estimation.

***Table S2.*** Sensitivity, specificity, negative and positive predictive value of p16/Ki-67 DS results for detecting CIN2+ in three cytopathology laboratories, for references1 and reference2 before and after the additional training.

| **Training** | **Laboratory** |  | **Sensitivity (95% CI), %** | **Specificity (95% CI), %** | **PPV (95% CI), %** | **NPV (95% CI), %** |
| --- | --- | --- | --- | --- | --- | --- |
| Initial | LAB1 |  | 82.5 (70.9-90.9) | 72.7 (60.4-83.0) | 74.3 (62.4-84.0) | 81.4 (69.1-90.3) |
|  | LAB2 |  | 90.5 (80.4-96.4) | 63.6 (50.9-75.1) | 70.4 (59.2-80.0) | 87.5 (74.8-95.3) |
|  | LAB3 |  | 79.4 (67.3-88.5) | 75.8 (63.6-85.5) | 75.8 (63.6-85.5) | 79.4 (67.3-88.5) |
| Addtional | LAB1 |  | 90.5 (80.4-96.4) | 75.8 (63.6-85.5) | 78.1 (66.9-86.9) | 89.3 (78.1-96.0) |
|  | LAB2 |  | 88.9 (78.4-95.4) | 68.2 (55.6-79.1) | 72.7 (61.4-82.3) | 86.5 (74.2-94.4) |
|  | LAB3 |  | 85.7 (74.6-93.3) | 75.8 (63.6-85.5) | 77.1 (65.6-86.3) | 84.7 (73.0-92.8) |
| Reference | Reference1 |  | 85.7 (74.6-93.3) | 72.7 (60.4-83.0) | 75.0 (63.4-84.5) | 84.2 (72.1-92.5) |
|  | Reference2 |  | 88.9 (78.4-95.4) | 71.2 (58.7-81.7) | 74.7 (63.3-84.0) | 87.0 (75.1-94.6) |

*PPV…positive predictive value, NPV…negative predictive value, LAB1…laboratory 1, LAB2…laboratory 2, LAB3…laboratory3, Reference1…consensus of 5 cytopathologists, Reference2…consensus obtained during discussion between participants of the study and expert.*
